# Dual effects of soil conditioners on wheat yield and soil properties in southern henan

**DOI:** 10.1038/s41598-026-55803-3

**Published:** 2026-06-05

**Authors:** Junhe Liu, Dongzhi Xu, Weina Zhang, Bingshen Jiang, Changli Liang, Han Wang, Hongmei Sun, Lianbin Cao

**Affiliations:** 1 School of Biological and Food Processing Engineering, Huanghuai University, Zhumadian, Henan China; 2 College of Life Science, Xinyang Normal University, Xinyang, Henan China

**Keywords:** Wheat, Soil conditioners, Soil characteristics, Microbial communities, Yield, Ecology, Ecology, Environmental sciences, Microbiology, Plant sciences

## Abstract

Aimed at addressing the constraints of farmland soil acidification on soil health and crop productivity in southern Henan Province, this study investigated the comprehensive ameliorative effects of different soil conditioners and identified the optimal amendment strategy. Based on a continuous fixed-point field experiment conducted from 2023 to 2025, treatments including NB (nitrogen fertilizer + biochar), NL (nitrogen fertilizer + lime), NM (nitrogen fertilizer + microbial fertilizer), NC (nitrogen fertilizer + calcium magnesium fertilizer), and controls were established. Crop yield, biomass, yield components, soil pH, ammonium nitrogen (NH₄⁺-N), nitrate nitrogen (NO₃⁻-N), available phosphorus (AP), available potassium (AK), soil organic carbon (SOC), aggregate size distribution, and rhizosphere bacterial and fungal communities were measured, and Spearman correlation analysis together was employed to link soil properties with microbial taxa and to reveal yield regulation pathways. The results indicated that the NB treatment exhibited the most pronounced comprehensive improvement: it achieved a grain yield of 8.38 t·ha⁻¹ (46.5% higher than the control), increased thousand grain weight by 12.3%, raised soil pH by 15.8% (from 3.49 to 4.04), and significantly increased soil NH₄⁺-N and NO₃⁻-N contents while maintaining stable available nitrogen supply, as well as significantly enhanced AP (40%), AK (85.6%), and SOC (33.1%). The proportion of macro- and meso- aggregates (0.25–2 mm) reached 19.06%, and fungal richness was notably increased, with key soil physicochemical indicators strongly correlated with specific microbial genera. Partial Least Squares Path Modeling (PLS-PM) further revealed that SOC exerted opposite regulatory effects on bacterial and fungal communities. The superior performance of NB is attributed to its multi pathway synergistic regulation—chemical pH buffering, physical structure improvement, nutrient retention especially for available nitrogen, and biological stimulation—achieving comprehensive and stable amelioration. Biochar combined with nitrogen fertilizer (NB) is the optimal amendment strategy for acidic soils in southern Henan, providing scientific evidence for the application of organic carbon conditioners in the management of acidified farmland.

## Introduction

Soil acidification has become a major environmental challenge to global agricultural sustainable development, and this issue is particularly prominent in China. Since the 1980s, the average pH of the topsoil in China’s major agricultural regions has decreased by 0.09–0.61 units^1^. During the same period, the area of acidic soils nationwide has expanded from approximately 3.0455 million km² during the Second National Soil Survey (1979) to the current 3.11 million km², accounting for 32.4% of the total land area, with a net increase of 64,500 km²^2^. Acidification not only significantly reduces the availability of key nutrients in the soil but also leads to an increase in exchangeable aluminum (Al³⁺) content, intensifying aluminum ion toxicity^3,4^. Furthermore, this process can activate heavy metal elements such as lead and cadmium, enhancing their bioavailability, while also suppressing soil microbial populations and related enzyme activities. The combined effects of these changes severely hinder crop growth and development, reduce yields, and may even lead to localized crop failures^5^.

The causes of soil acidification are complex, among which excessive application of nitrogen fertilizer (especially urea) is widely recognized as a primary anthropogenic driving factor. Nitrogen fertilizers undergo microbially driven mineralization and nitrification in the soil, continuously releasing ammonium ions, nitrate, and accompanying protons (H⁺), significantly increasing the hydrogen ion concentration in the soil solution and thereby accelerating the decline in pH. This process not only directly induces acidification but also leads to the leaching of base cations and nutrient imbalances, forming a vicious cycle of “aggravated acidification → nutrient depletion → reduced buffering capacity → further acidification,” continuously amplifying environmental risks in agricultural ecosystems^6^. It is noteworthy that once this cycle is established, it exhibits a self-reinforcing trend, making remediation significantly more challenging. In addition to fertilization, climatic conditions (such as intense leaching in high-rainfall regions), inherent soil properties (such as differences in clay content and buffering capacity), and unreasonable farming practices also exacerbate the acidification process from various dimensions.

In China, soil acidification progresses more rapidly and severely in the humid southern regions, becoming a key obstacle to high-quality regional agricultural development. Taking the wheat planting area in southern Henan as an example, long-term intensive production and excessive input of chemical nitrogen fertilizers have led to a significant acidification trend in this region, with the pH of some farmland dropping below 5.5. This severely hinders crop root development and nutrient uptake, directly threatening grain production capacity and quality. As acidification continues to develop, the accelerated loss of available soil nutrients, increased toxicity of aluminum and manganese ions, and elevated heavy metal activity not only lead to soil fertility decline but may also pose health risks through the food chain, constituting ongoing pressure on China’s agricultural and ecological security.

Faced with this severe situation, the rational application of soil amendments has become an important approach to remediate acidified soils and restore soil fertility. Amendments can comprehensively improve soil physicochemical properties, mitigate degradation trends, and enhance soil biological activity and overall fertility by adjusting pH and optimizing aggregate structure. For example, materials such as biochar and lime can significantly promote the yield of crops like wheat by increasing soil pH and improving the micro-environment for crop growth. Biochar, derived from straw pyrolysis, is rich in carboxyl groups and alkaline. When applied to acidified soil, its carboxyl groups can bind with hydrogen ions, reducing acidity and alleviating aluminum toxicity. However, Huang et al.^7^ point out that while traditional inorganic amendments like lime can rapidly increase pH, single applications often lack long-lasting effects. Therefore, microbial fertilizer-based amendments have gradually become a research focus in recent years. These products introduce beneficial microorganisms to regulate microbial community structure and enhance species diversity in acidic soils, thereby promoting nutrient cycling and improving crop stress resistance. Zhang et al.^8^ reported that microbial fertilizers can significantly reduce soil nitrate levels, decrease toxic nitrite accumulation, increase ammonium nitrogen content, and improve soil fertility and remediation effects. However, Liu et al.^9^ also found that not all amendments promote microbial activity; some products may even inhibit it, leading to declines in soil fertility and yield. Overall, although existing research preliminarily demonstrates the potential of soil amendments and their broad application prospects in acidic soil remediation and sustainable agricultural production, we must recognize that systematic, long-term field validation based on regional soil characteristics is indispensable for refining product formulations and application techniques.

To this end, based on the practical needs of acidified farmland in southern Henan, this study conducted a two-year field experiment to systematically investigate the dynamic regulatory effects of four amendments—lime, biochar, microbial fertilizer, and silicon-potassium fertilizer—on soil acidity, and to validate the actual impact of the amended soil on wheat growth. The aim is to clarify the effects of different types of soil conditioners (applied alone and in combination with nitrogen fertilizer) on soil pH improvement and wheat yield in acidic soils, ultimately providing a reliable scientific basis for the targeted management of regional acidified soils.

## Materials and methods

### Experimental site, soil, and climatic conditions

**Fig. 1:**
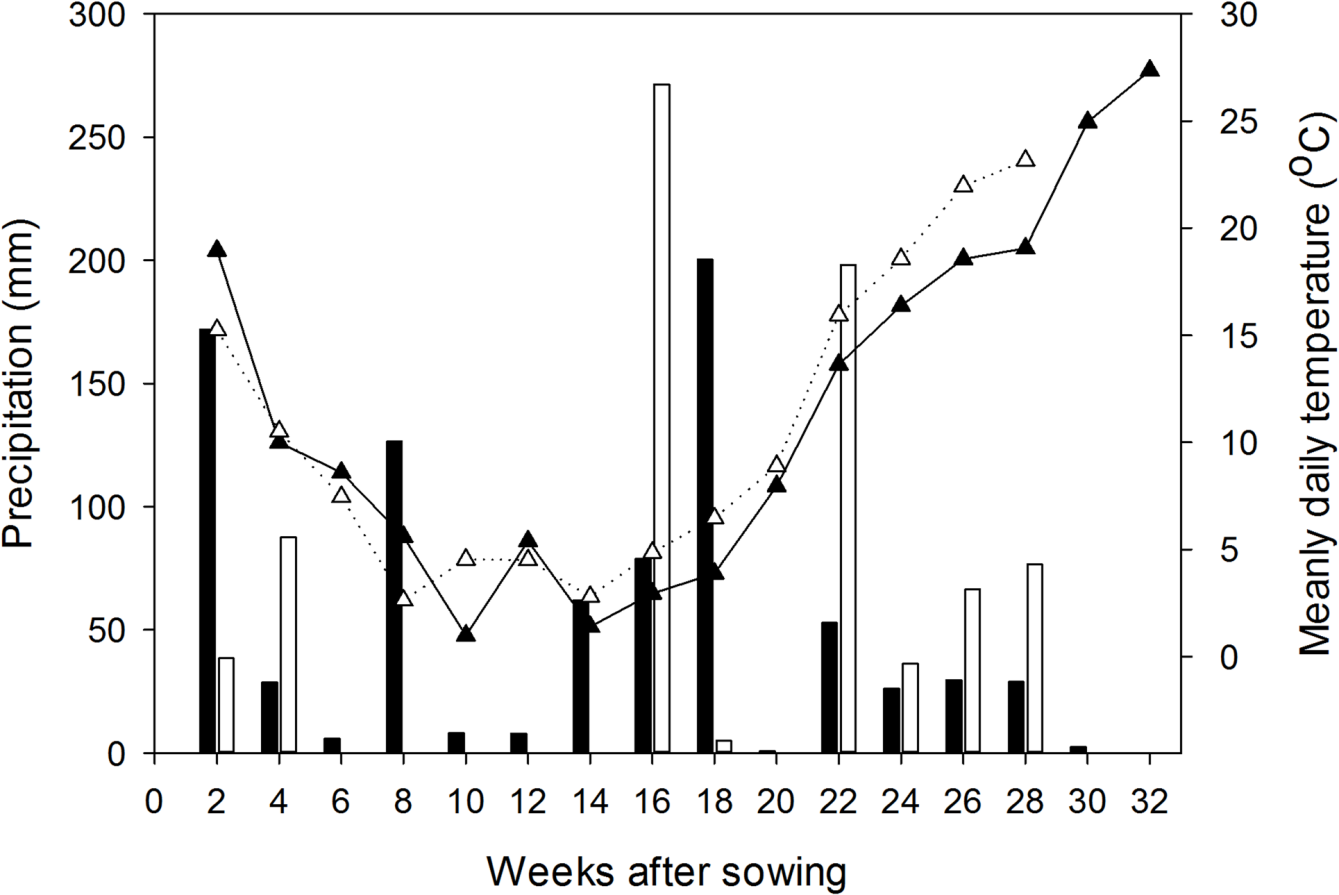
Weekly precipitation (bars), mean daily temperature (triangles) during wheat growing season in 2023–2024 (closed symbols) and 2024–2025 (open symbols). 、.

A two-year field experiment was conducted in Shahedian Experimental Station of Huanghuai University (33°12′N, 114°03′E) from 2023 to 2025 to explore the acidification improvement effects of different types of soil conditioners on acidic soils. This study was conducted as a continuous two-year field experiment on the same plots, with fixed plot positions for each treatment. The experimental site is located in the southern part of the North China Plain—a major grain-producing area in China—and the soil type is yellow cinnamon soil developed from Quaternary Loess (Q₃) parent material. The physical and chemical properties of the initial surface soil (0–20 cm) during the wheat sowing period in 2023 were: pH (H₂O, 1:2.5 w/v) 3.5, Total Nitrogen 0.53 g kg⁻¹, organic matter content 12.43 g kg⁻¹, available potassium 74 mg kg⁻¹, and available phosphorus 15.0 mg kg⁻¹. Meteorological parameters (including weekly precipitation and daily average temperature) are shown in Fig. 1. The total precipitation during the experimental period (October 2023 to May 2025) reached 1028.7 mm. Only wheat was cultivated during the experimental period, with the maize season left fallow。.

### Experimental design

A two-factor randomized block design was adopted in our study, with two nitrogen application levels (0 kg·ha^− 1^ and 225 kg·ha^− 1^) and five types of soil conditioners (none, lime, biochar, microbial fertilizer, and calcium-magnesium fertilizer), forming a total of 10 treatment combinations (Table 1). The composition, pH, and manufacturer of the conditioners are shown in Table 2. Each treatment had 4 replicates, and the plot area was 30 m² (6 m × 5 m). All plots were uniformly applied with phosphorus fertilizer (superphosphate, containing 16% P₂O₅) at 75 kg· P₂O₅·ha-1 and potassium fertilizer (potassium sulfate, containing 50% K₂O) at 60 kg·K₂O·ha-1. Nitrogen fertilizer was urea (containing 46% N). Phosphorus and potassium fertilizers were applied as base fertilizers and ploughed into the 0–20 cm soil layer before sowing; nitrogen fertilizer was split-applied at a base-topdressing ratio of 6:4, with 60% as base fertilizer and 40% top-dressed during the jointing stage combined with rainfall (under rain-fed conditions). The winter wheat variety was ‘Suimai 139’ (SM 139), sown on October 25, 2023, and November 1, 2024, respectively, with a sowing rate of 525 kg·ha^− 1^. Seeds of SM 139 were purchased from Zhumadian Yufeng Seed Industry Co., Ltd. (Zhumadian, China), requiring no collection permits as a commercial crop. A voucher specimen (No. SM139-2026-01) was deposited in the Herbarium of Huanghuai University and authenticated by Prof. Junhe Liu. Weeds and pests were controlled, herbicides were sprayed after jointing and tillering growth stage. Field management followed the high-yield cultivation model, and fertilizers were uniformly mixed into the 0–30 cm plough layer using a rotary tiller before sowing.

**Table 1:** Application rates of nitrogen fertilizer and soil amendments for each treatment.

Treatment	Amendment Type	*N* Application Rate (kg·ha⁻¹)	Amendment Application Rate (kg·ha⁻¹)
Control	None	0	0
Urea	None	225	0
Lime	Lime	0	450
Biochar	Biochar	0	6000
MF	Microbial fertilizer	0	150
Ca-Mg	Calcium-magnesium fertilizer	0	300
NL	Urea + Lime	225	450
NB	Urea + Biochar	225	6000
NM	Urea + Microbial	225	150
NC	Urea + Calcium-magnesium fertilizer	225	300

**Table 2:** Composition, pH value, and manufacturer of the conditioners.

Conditioner	Composition	pH	Manufacturer
Lime	Effective CaO content ≥ 94% after calcination	9.0	Henan Jinfeng Environmental Protection Engineering Co., Ltd.
Biochar	Rice straw, pyrolyzed at 400–600 °C, carbon content ≥ 78%	10.5	Zhengzhou Jinbang Environmental Protection Technology Co., Ltd.
MF	Mainly contains actinomycetes, yeast, and phosphate-solubilizing Bacillus, etc.	8.5	Zhumadian Agricultural Association
Ca-Mg	MgO ≥ 25%, CaO ≥ 16%, activated from natural ore materials	9.0	Sino-Agri Leading Biosciences Co., Ltd.

### Sample Collection and Analysis

At wheat maturity, 20 winter wheat stems were randomly collected from each plot. The stems were cut at the soil surface to determine plant height, leaf area, and aboveground biomass, and plant density was recorded concurrently. The leaf area of fully expanded leaves was calculated by the formula: measured leaf length × measured leaf width × 0.75. Plant samples were held at 105 °C for 30 min and then dried at 70 °C to a constant weight. At harvest, wheat plants in the central 1 m² area of each plot were harvested, and grains were separated using a thresher. Grain weight was measured after natural air-drying to a constant moisture content of 13%. Soil samples were collected at maturity growth stage of wheat. Five soil cores (0–20 cm depth) were randomly obtained from each sampling point. Samples were divided into two parts: one part was air-dried in a cool and well-ventilated place, and after removing plant residues, weed roots, and gravel, it was sieved through a 20-mesh sieve for the analysis of soil pH, Olsen-P, and available potassium; the other part was stored in a -80 °C ultra-low temperature refrigerator for subsequent analysis of soil microbial indicators.

The determination of soil physical and chemical properties referred to the conventional methods in Soil and Agricultural Chemistry Analysis^10^. Sieved soil samples (2 mm) were used to determine pH (soil-water ratio 1:2.5, w/v), available phosphorus (Olsen-P method, NaHCO₃ extraction), and available potassium (NH₄OAc extraction). Air-dried soil samples were used to determine soil organic carbon (SOC). Ball-milled samples were pretreated with 0.5 mol L^− 1^ HCl to remove carbonates and rinsed with deionized water. SOC of original soil and dry-sieved fractionated samples was determined by the dry combustion method using an elemental analyzer (Vario Macro CNS, Elementar, Germany) after acid washing. Dry sieving and wet sieving methods were used to separate aggregates of different particle sizes. The brief procedure is as follows: 200 g of pre-sieved (8 mm) soil sample was sequentially passed through 2.0 mm and 0.25 mm sieves to obtain three fractions: >2.0 mm (macro-aggregates), 0.25–2.0 mm (micro-aggregates), and < 0.25 mm (micro-aggregates and silt-clay). Subsequently, the < 0.25 mm fraction was further passed through a 0.053 mm sieve to separate it into 0.053–0.25 mm (micro-aggregates) and < 0.053 mm (silt-clay fraction). After sieving, the samples were oscillated in an aggregate analyzer (DM185, Shanghai Dima Industry) with a circular motion at 1450 rpm for 2 min. Fractionated aggregates were divided into two parts: one part was air-dried to determine aggregate-associated organic carbon, and the other part was used for wet sieving analysis. For wet sieving, 30 g of soil was taken, and the fractionation standards were the same as dry sieving. Nested sieves were placed in a bucket connected to a motor, soil was evenly spread on the top sieve, immersed in deionized water for 3 min, and then lifted and lowered at a rate of 30 times minute^− 1^ for 4.0 cm for 2 min. Floating organic matter was discarded, and aggregates retained on each sieve were transferred to containers, dried in a vacuum oven at 65 °C for 48 h, and then weighed. The soil recovery rate of wet sieving was > 90%. The water stability of aggregates was expressed as the mean weight diameter (MWD, mm), calculated by the formula (van Bavel, 1950):

MWD = Σ (d × m).

Where d is the average diameter of adjacent sieves (mm), and m is the mass fraction of aggregates on the sieve (%).

### High-throughput sequencing and soil microbial analysis

To explore the microbial community structure under different fertilization treatments high-throughput sequencing of the 16 S rRNA and fungal ITS regions was performed. DNA extraction, purification, PCR amplification, and library construction were completed by Shanghai Majorbio Biomedical Technology Co., Ltd. The V3-V4 region of the bacterial 16 S rRNA gene was amplified using primers 338 F (5’-ACTCCTACGGGAGGCAGCA-3’) and 806R (5’-GGACTACHVGGGTWTCTAAT-3’); the fungal ITS1 region was amplified using primers ITS1F (5’-CTTGGTCATTTAGAGGAAGTAA-3’) and ITS2R (5’-GCTGCGTTCTTCATCGATGC-3’). Qualified sequences were clustered into OTUs at 97% similarity using UPARSE v7.1 software, and chimeras were removed. Sequences derived from chloroplasts and mitochondria in all samples were filtered out. OTU taxonomic annotation was performed by comparing with the SILVA 16 S rRNA database and fungal ITS database using RDP classifier (confidence threshold 70%), and community composition was counted at different taxonomic levels. All microbial data analyses were completed on the Majorbio Cloud Platform (https://cloud.majorbio.com*).*

### Statistical analysis

Data management was performed using Microsoft Excel 2007. Statistical analysis was conducted using analysis of variance (ANOVA) with SPSS software (version 20.0, 2018), and post-hoc tests were performed using the least significant difference (LSD) method at the *P* < 0.05 level. All Figs were drawn using Origin software.

## Results

### Effects of different soil conditioners on yield and yield components of wheat

Based on a two-year continuous field experiment (Table 3), different soil conditioner treatments significantly affected wheat grain yield, aboveground biomass, and yield components, and these effects were co-regulated by nitrogen application rate and interannual factors. As the nitrogen application rate increased from 0 to 225 kg·ha⁻¹ combined with conditioners such as biochar (NB) or lime (NL), grain yield and aboveground biomass increased accordingly; when yield reached 8–8.5 t·ha⁻¹, it stabilized and no longer increased significantly with further conditioner combinations. Regardless of nitrogen application, the harvest index (grain yield/aboveground biomass) remained between 0.4 and 0.5. From the control treatment to the combined amendment treatments with nitrogen fertilizer (NB, NL), spike number per hectare at wheat maturity increased with treatment intensity and stabilized at 3.83 × 10⁶–3.95 × 10⁶ spikes·ha⁻¹. Spike number per spike increased significantly and then remained stable after switching from the control to NB or NL treatments. After thousand-kernel weight reached approximately 38 g, it increased slightly rather than decreasing with further optimization of amendments (e.g., from NL to NB), reaching 38.78 g in the NB treatment and 37.52 g in the NL treatment.

**Table 3:** Effects of different soil conditioners and nitrogen application rates on wheat grain yield, biomass, and yield components (2023–2024 and 2024–2025).

Year	Treatment		Yield(t ha^− 1^)	Biomass (t ha^− 1^)	Spike number per ha (10^4)	Kernels per spike	Thousand kernel weight (g)
	*N* rate (kg ha^− 1^)	Soil conditioner type					
2023–2024	0	None	5.60ef	17.85 cd	338f	36.6d	35.4a
		Lime	6.60 cd	18.6 cd	391 cd	42.9ab	30.8bc
		Biochar	6.90 cd	18.9bc	405bc	42.3bc	31.5bc
		MF	6.24de	18.1 cd	377de	41.0bc	31.5bc
		Ca-Mg	6.15de	18.4 cd	372de	39.4bc	32.8bc
	225	None	5.26f	17.1d	355de	37.7 cd	30.7c
		Lime	7.93ab	19.6ab	420ab	45.1ab	32.5b
		Biochar	8.12a	20.01a	431a	46.2a	31.9b
		MF	7.65ab	18.7bc	415bc	43.8ab	33.0b
		Ca-Mg	7.32bc	18.9ba	408bc	43.7ab	32.1b
2024–2025	0	None	5.72ef	18.5bc	327e	39.7bc	34.5 cd
		Lime	6.61 cd	19.0bc	343bc	43.1ab	35.4 cd
		Biochar	6.86 cd	19.2bc	340bc	43.5ab	36.7bc
		MF	6.27de	18.8bc	339 cd	40.2bc	36.1 cd
		Ca-Mg	6.22de	18.9bc	343bc	41.7ab	34.2 cd
	225	None	5.18f	17.4c	332 cd	38.6bc	31.7e
		Lime	8.11ab	21.5ab	395a	42.8ab	37.5ab
		Biochar	8.38a	21.9a	383ab	44.3ab	38.7a
		MF	7.77bc	20.1ab	385ab	43.5ab	36.5bc
		Ca-Mg	7.51bc	20.3ab	355bc	44.8a	37.1bc
Source of variation	N application rate (N)		***	***	***	**	ns
	Soil conditioner type (C)		***	***	***	***	ns
	N * C		***	***	ns	ns	***

All these parameters differed significantly between growing seasons. Wheat yield, biomass, and spike number in the 2024–2025 growing season were much higher than those in the 2023–2024 season, confirming the long-term cumulative effects of the conditioners. Moreover, Two way ANOVA showed that nitrogen application rate (N), conditioner type (C), and their interaction (N × C) all had highly significant effects on wheat yield and biomass (*P* < 0.001). Nitrogen application rate significantly affected spike number per hectare, while conditioner type mainly regulated kernels per spike. The N × C interaction had a highly significant effect on thousand-kernel weight but no significant effect on spike number per hectare or kernels per spike.

### Effects of different soil conditioners on soil physiochemical properties of wheat rhizosphere soil

**Fig. 2:**
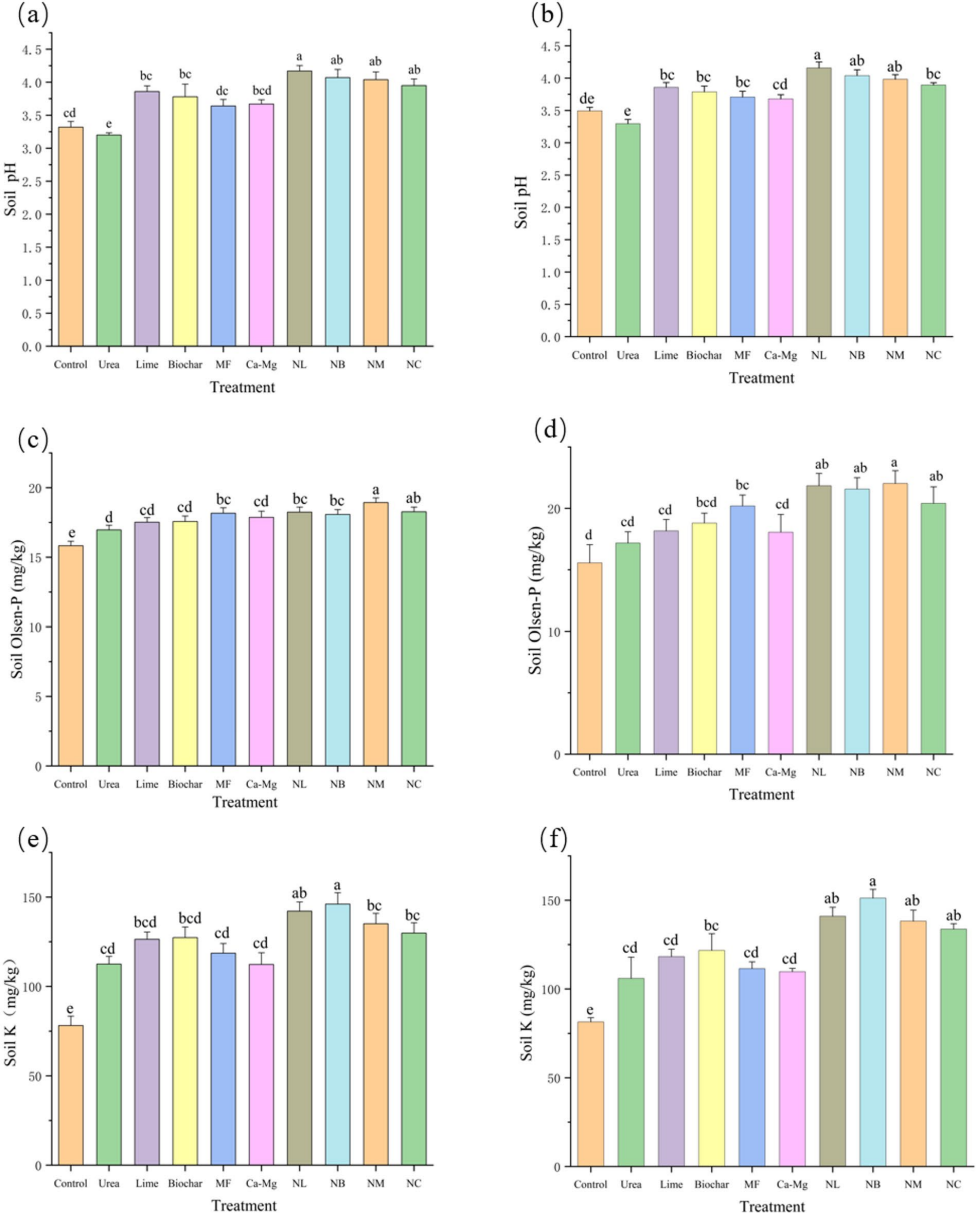
Effects of soil conditioners on soil pH (a, b), available phosphorus (c, d), and available potassium (e, f) in 2024 and 2025. Error bars represent the standard error of the mean of 4 replicates (*n* = 4). Different lowercase letters indicate significant differences between treatments (*P* < 0.05).

Based on continuous in-situ observations from 2024 to 2025 (Fig. 2), different soil improvement measures showed significant and stable hierarchical differences in their effects on key chemical indicators of acidic soil, which can be generally divided into three categories. In terms of soil pH, the nitrogen fertilizer + lime (NL) and nitrogen fertilizer + biochar (NB) treatments exhibited excellent acid-adjusting effects. By 2025, the NL treatment increased soil pH to 4.16, a 19.2% improvement compared with the control (3.49), and the NB treatment reached pH 4.04, an increase of 15.8%, significantly alleviated soil acidification. In contrast, the urea-only treatment had the lowest pH (3.30), a further 5.4% decrease from the control, significantly exacerbating soil acidification. The interannual fluctuation of pH in all treatments was generally less than ± 0.05, indicating that the acid-regulating effect stabilized within the first year after application. For available phosphorus (AP), the nitrogen fertilizer + microbial fertilizer (NM) treatment showed the most significant increase, reaching 22.03 in 2025, a 41.5% rise over the control (15.56). The NL (21.84) and NB (21.58) treatments also increased by nearly 40%, demonstrating strong phosphorus activation and supply capacity. Although the urea-only treatment (17.18) was 10.4% higher than the control, it remained significantly lower than all combined treatments. Moreover, available phosphorus in superior treatments such as NL continued to accumulate in the second year, with an annual increase of 19.8%, showing a sustained nutrient activation effect.

In terms of available potassium (AK), the NB treatment performed most prominently, reaching 151.25 in 2025, an 85.6% surge compared with the control (81.5), and the NL treatment (141.00) also increased by 73.0%. Conversely, the available potassium content in the urea-only treatment (106.00) decreased by 5.8% in the second year, showing a trend of soil potassium pool depletion. Overall, the NB and NL treatments achieved synergistic improvements in multiple key soil chemical indicators, forming the optimal improvement group by simultaneously regulating acidity, increasing phosphorus, and stabilizing potassium. Traditional single amendments (e.g., lime, biochar) had limited effects, with only moderate improvements in individual indicators (e.g., lime increased pH by 10.6%). The urea-only treatment was the only one with systematic negative effects, aggravating soil acidification and depleting nutrient pools. In terms of interannual dynamics, the soil pH improvement effect was highly temporally stable, while available phosphorus and potassium in superior treatments showed a continuous cumulative upward trend, which was highly consistent with the interannual changes in crop yield and biomass.

**Fig. 3:**
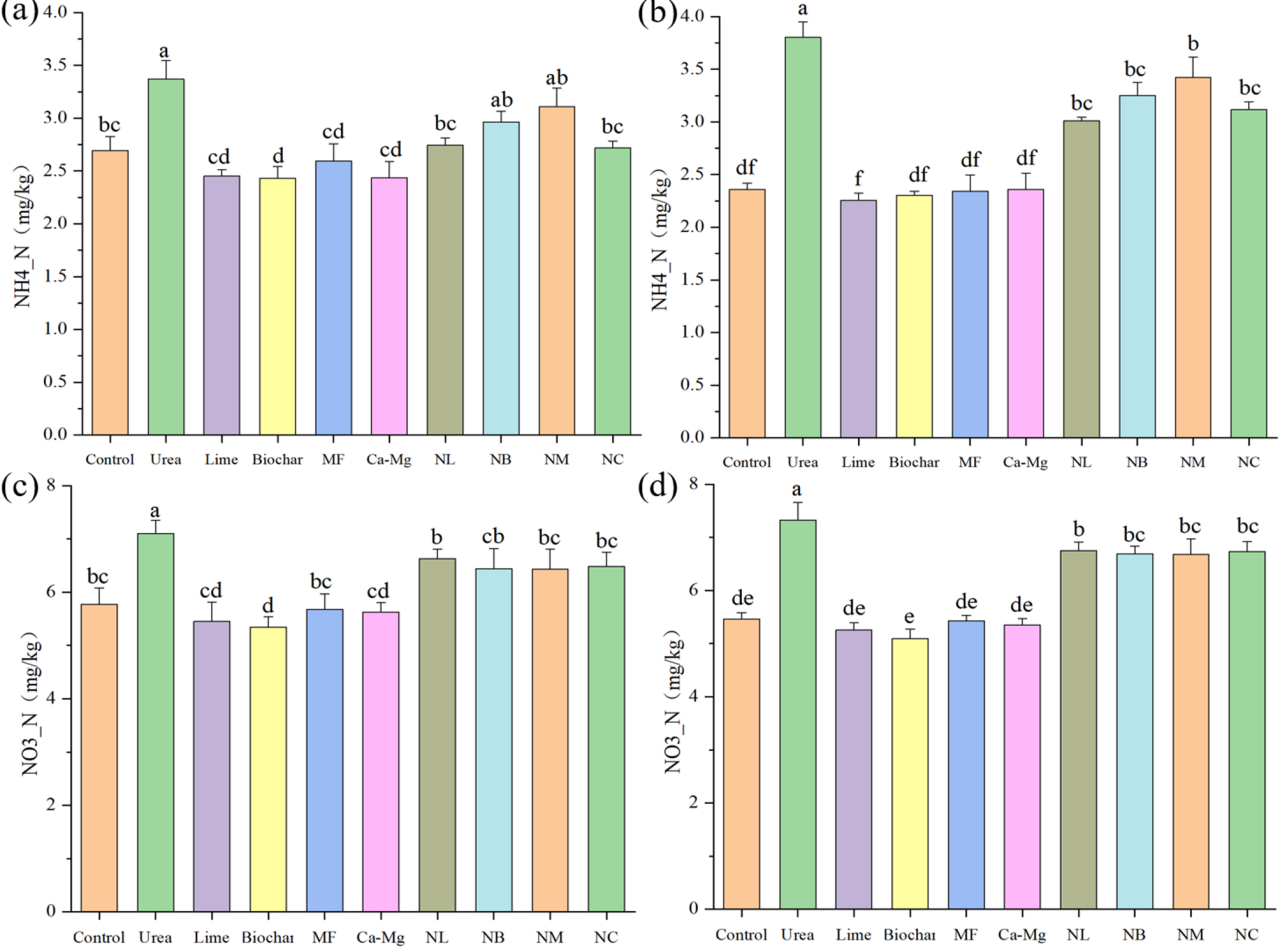
Effects of soil conditioners on soil NH4_N (a, b), NO3_N (c, d) in 2024 and 2025. Error bars represent the standard error of the mean of 4 replicates (*n* = 4). Different lowercase letters indicate significant differences between treatments (*P* < 0.05).

The effects of different fertilization and soil amendment treatments on soil ammonium nitrogen (NH₄⁺-N) and nitrate nitrogen (NO₃⁻-N) contents from 2024 to 2025 are shown in Fig. 3. Consistent trends were observed across the two years. The urea (Urea) treatment significantly increased both NH₄⁺-N and NO₃⁻-N contents compared to all other treatments (*P* < 0.05), reaching the highest levels overall: NH₄⁺-N contents were approximately 3.4 mg/kg and 3.8 mg/kg in 2024 and 2025, respectively, while NO₃⁻-N contents were about 7.0 mg/kg and 7.5 mg/kg. The sole applications of lime (Lime), biochar (Biochar), medium and trace element fertilizer (MF), and calcium-magnesium fertilizer (Ca-Mg) showed significantly weaker effects on enhancing soil available nitrogen than urea. Specifically, the Biochar treatment resulted in the lowest NH₄⁺-N content across all treatments in both years, and the Lime and Biochar treatments also exhibited relatively low NO₃⁻-N contents. Compared with the Urea treatment, the combined treatments of nitrogen fertilizer with soil amendments (NL, NB, NM, NC) maintained high levels of both NH₄⁺-N and NO₃⁻-N throughout the two-year experiment: the NM treatment showed no significant difference in NH₄⁺-N content from the Urea treatment in 2024 (*P* > 0.05); the NO₃⁻-N contents of NL, NB, NM, and NC were significantly higher than those of the control and single amendment treatments (*P* < 0.05); in 2025, although the NO₃⁻-N contents of these combined treatments were significantly lower than that of the Urea treatment, they still fell within the same significance group.

**Fig. 4:**
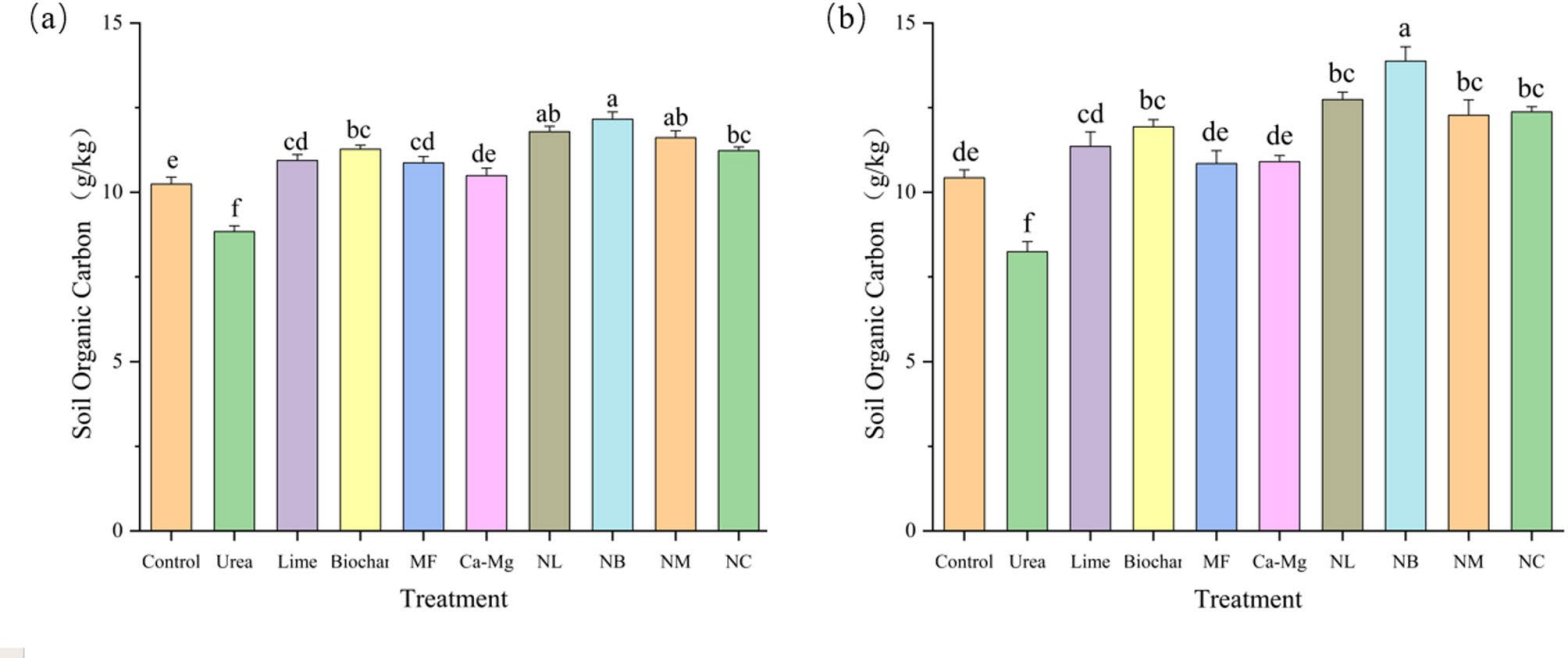
Effects of soil conditioners on soil organic carbon (SOC) in 2024 (a) and 2025 (b). Error bars represent the standard error of the mean of 4 replicates (*n* = 4). Different lowercase letters indicate significant differences between treatments (*P* < 0.05).

Soil organic carbon (SOC) is a core indicator for maintaining soil quality and farmland carbon sequestration. Based on continuous in-situ observations from 2024 to 2025 (Fig. 4), different improvement treatments exerted highly significant and stratified effects on SOC content. The nitrogen fertilizer combined with biochar or lime (NB, NL) treatments performed best in enhancing soil carbon sequestration and fertility. By 2025, their SOC contents reached 13.88 and 12.74, representing increases of 33.1% and 22.1% over the control (10.43), respectively, with obvious advantages. In sharp contrast, the urea-only treatment caused a net loss of the soil carbon pool, with an SOC content of 8.24, 21.0% lower than the control, and a further 6.7% decline over the two years.From interannual changes, the NB and NL treatments displayed strong and sustained carbon sequestration capacity, with SOC contents increasing significantly by 14.1% and 8.1% in the second year, respectively. Treatments combining nitrogen fertilizer with microbial fertilizer or calcium-magnesium fertilizer (NM, NC), as well as traditional single amendments (biochar, lime), also showed stable carbon-increasing effects.

**Fig. 5:**
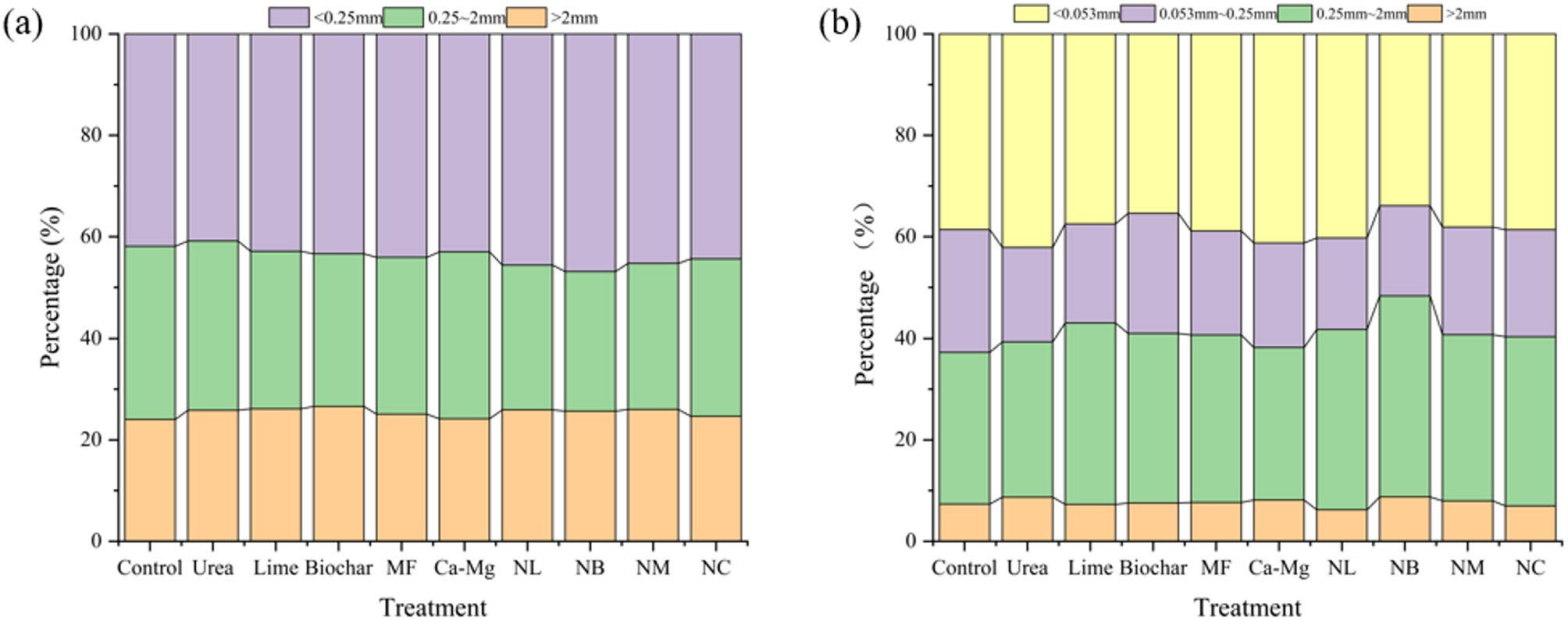
Effects of soil conditioners on soil aggregates fractionation in 2024 (a) and 2025 (b). Error bars represent the standard error of the mean of 4 replicates (*n* = 4). Different lowercase letters indicate significant differences between treatments (*P* < 0.05).

Similarly, the continuous observation data of soil aggregate distribution from 2024 to 2025 (Fig. 5) show that, different improvement treatments differed significantly in optimizing soil structure. The combined treatments of nitrogen fertilizer + biochar (NB) and nitrogen fertilizer + lime (NL) performed best and effectively promoted aggregate formation and structural stability. In 2024, the > 2 mm macroaggregate content in the NB treatment was significantly higher than in the control and urea-only treatment. Although the proportion of macroaggregates decreased across all treatments in 2025, NB and NL maintained a significant advantage in the key 0.25–2 mm fraction. The urea-only treatment showed obvious soil structural degradation, with the highest silt-clay fraction. Interannual variations revealed that the decline in > 2 mm macroaggregates was mainly related to the attenuation of the initial physical effects of amendments and spatiotemporal mismatch of biological cementation. However, NB and NL delayed this process by increasing soil pH and improving microbial habitat, thus maintaining favorable soil structure.

### Effects of different soil conditioners on microbiome composition and relative abundance

**Fig. 6:**
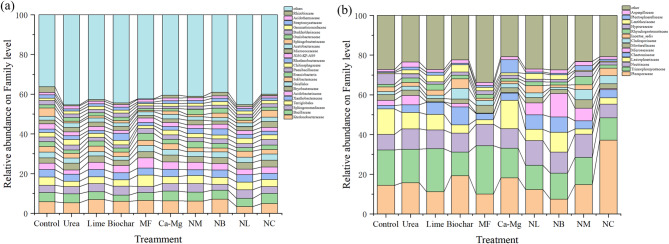
Community composition of bacteria (a) and fungi (b) at the family level under different treatments.

Results of alpha diversity analysis of the microbial community in the wheat rhizosphere showed significant differences in the responses of bacterial and fungal communities to soil conditioners. The effects of soil conditioners on the richness and diversity of the bacterial community were detectable but relatively mild overall (Table 4). Chao1 and ACE indices indicated that lime, biochar, calcium magnesium fertilizer, and the NB treatment tended to increase bacterial richness, with the lime treatment showing the highest Chao1 (3576.8) and ACE (3572.6) values. In contrast, the NM treatment exhibited the lowest richness, suggesting a potential inhibitory effect on bacterial community size. Despite these differences in richness, Shannon and Simpson indices did not differ statistically among treatments, indicating that the overall diversity and evenness of the bacterial community remained relatively stable, and its core diversity structure showed strong resilience to soil conditioners. The coverage values of all treatments reached 98%, confirming sufficient sequencing depth to reliably represent the actual community composition. In comparison, the alpha diversity of the rhizosphere fungal community showed clear treatment-dependent responses to different soil conditioners (Table 5). Chao1 and ACE indices revealed that lime significantly increased fungal richness and performed best among all treatments; NB and biochar also induced moderate increases, whereas the NM treatment consistently showed the lowest richness, implying potential suppression of certain fungal taxa. Diversity indices further revealed significant variations: the Shannon indices were highest under lime, MF, and calcium magnesium fertilizer treatments, indicating more diverse and evenly distributed fungal communities. Conversely, urea and NM treatments tended to reduce community evenness, as reflected by their higher Simpson values. All treatments achieved a consistently high coverage of 99%, verifying adequate sequencing depth. In summary, the core diversity structure of the rhizosphere bacterial community was highly stable in response to soil conditioners, whereas the fungal community was more sensitive. Among all treatments, lime exerted the strongest positive regulatory effect on fungal richness and diversity. A systematic analysis of the microbial community under different soil conditioner treatments (Fig. 6) revealed that in the bacterial community, unclassified bacteria dominated, with a relative abundance ranging from 36.2% to 45.3%, reaching the highest value (45.3%) under urea treatment. The core dominant groups were *Ktedonobacteraceae* and *Bacillaceae* (> 3%). Lime treatment enriched *Ktedonobacteraceae* (6.90%) and *Acidobacteriaceae* (3.71%); microbial fertilizer (MF) promoted *Terriglobales* (5.67%) and *Acidobacteriaceae* (5.16%); calcium-magnesium fertilizer increased Bacillaceae to 4.87%; urea inhibited *Acetobacteraceae* (0.90%) and *Acidothermaceae* (0.45%). *Bacillaceae* and *Sphingomonadaceae* exhibited strong environmental adaptability, while *Acetobacteraceae* and *Acidothermaceae* showed significant abundance fluctuations and could serve as potential indicators of soil environmental changes. Among combined conditioners, NL enriched *Sphingomonadaceae* (4.39%), while NC promoted balanced development of *Bacillaceae* (5.16%) and *Acetobacteraceae* (3.40%). Regarding the fungal community, the relative abundance of unclassified fungi ranged from 20.77% to 33.72%, peaking under MF treatment. The core dominant groups were *Trimorphomycetaceae*, *Pleosporaceae*, and *Nectriaceae*, each exceeding 10% in most treatments. MF specifically enriched *Trimorphomycetaceae* (24.34%); NC extremely enriched *Pleosporaceae* (37.13%) while significantly suppressing *Trimorphomycetaceae. Pleosporaceae*, *Trimorphomycetaceae*, and *Microascaceae* were sensitive to environmental changes, whereas *Mortierellaceae*, *Cladosporiaceae*, and *Aspergillaceae* remained stable in abundance, constituting the core groups maintaining functional stability of the fungal community.

**Table 4:** Effect of different soil conditioners on Alpha diversity of bacterial communities in wheat rhizosphere soil.

Treatments	Chao1 Index		ACE Index	Shannon Index	Simpson Index	Coverage (%)
Control		3170.4 ± 117.5 ab	3145.9 ± 111.4 b	6.3 ± 0.04 a	0.004 ± 0.00 a	98 a
Urea		3230.1 ± 150.5 ab	3210.1 ± 155.8 ab	6.4 ± 0.08 a	0.004 ± 0.00 a	98 a
Lime		3576.8 ± 93.96 a	3572.6 ± 89.9 a	6.4 ± 0.07 a	0.004 ± 0.00 a	98 a
Biochar		3337.4 ± 134.6 ab	3313.2 ± 130.5 ab	6.3 ± 0.06 a	0.004 ± 0.00 a	98 a
MF		3329.5 ± 121.9 ab	3306 ± 126.6 ab	6.3 ± 0.06 a	0.004 ± 0.00 a	98 a
Ca-Mg		3359.4 ± 131.8 ab	3346.3 ± 118 ab	6.4 ± 0.04 a	0.004 ± 0.00 a	98 a
NL		3206.9 ± 202.1 ab	3186.8 ± 203 ab	6.3 ± 0.08 a	0.004 ± 0.00 a	98 a
NB		3337 ± 62.36 ab	3337.4 ± 60.1 ab	6.4 ± 0.04 a	0.004 ± 0.00 a	98 a
NM		2980.8 ± 79.63 b	2973.5 ± 75.86 b	6.3 ± 0.03 a	0.004 ± 0.00 a	98 a
NC		3236.7 ± 118.9 ab	3227.8 ± 126.4 ab	6.3 ± 0.05 a	0.004 ± 0.00 a	98 a

**Table 5:** Effect of different soil conditioners on Alpha diversity of fungal communities in wheat rhizosphere soil.

Treatments	Chao1 Index	ACE Index	Shannon Index	Simpson Index	Coverage (%)
Control	486 ± 17 ab	480.2 ± 13.5 ab	3.4 ± 0.15 b	0.1 ± 0.01 a	99 a
Urea	500.2 ± 30.8 ab	500.2 ± 32.8 ab	3.4 ± 0.19 ab	0.08 ± 0.01 abc	99 a
Lime	588.2 ± 30.5 a	590 ± 27.6 a	3.9 ± 0.06 a	0.04 ± 0.00 c	99 a
Biochar	522.5 ± 8.82 ab	525.9 ± 11 ab	3.8 ± 0.05 ab	0.05 ± 0.00 bc	99 a
MF	529.2 ± 12.9 ab	530.7 ± 11.9 ab	3.7 ± 0.14 ab	0.05 ± 0.01 bc	99 a
Ca-Mg	549.5 ± 14.7 ab	542.3 ± 10.9 ab	3.5 ± 0.16 ab	0.08 ± 0.01 abc	99 a
NL	520.2 ± 59.6 ab	515.7 ± 59.4 ab	3.8 ± 0.11 ab	0.06 ± 0.01 abc	99 a
NB	562.4 ± 28.3 ab	561.5 ± 29.1 ab	3.4 ± 0.2 b	0.1 ± 0.02 ab	99 a
NM	516.1 ± 18.2 b	516.5 ± 20.3 b	3.7 ± 0.21 ab	0.06 ± 0.01 abc	99 a
NC	524.7 ± 12.5 ab	528.7 ± 11.3 ab	3.8 ± 0.08 ab	0.05 ± 0.00 c	99 a

**Fig. 7:**
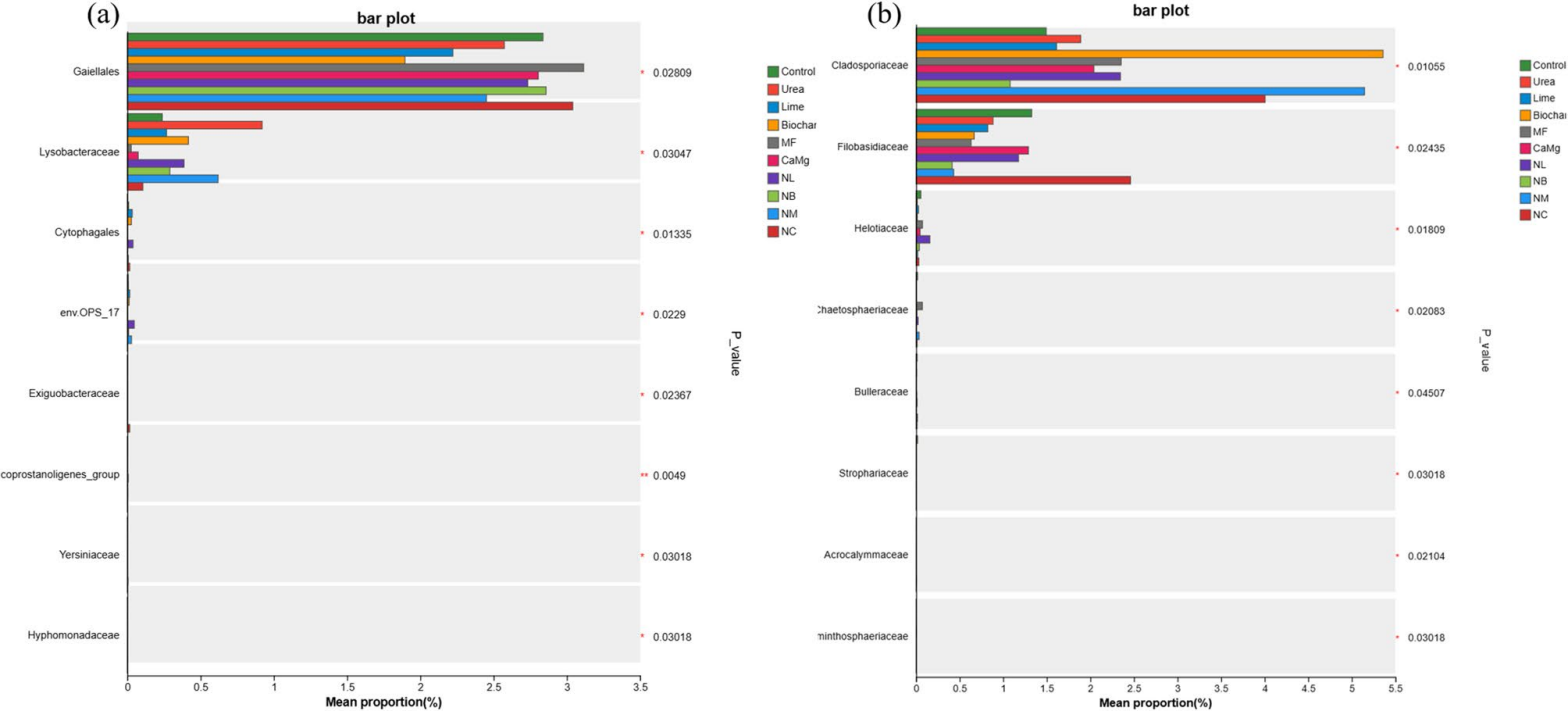
Relative abundance of bacterial (a) and fungal (b) communities under different treatments.

Significant test results of inter-group differences in the soil microbiome (Fig. 7A) revealed distinct differentiation patterns in the composition of bacterial communities across treatments: the relative abundance of *Gaiellales* was significantly higher under NB and NC treatments than in other groups, with an average relative abundance close to 3%; *Lysobacteraceae* exhibited a unique enrichment pattern, with its relative abundance ratio in the urea-only treatment being significantly higher than in all other treatments; the relative abundance of *Cytophagales* was generally below 0.5% but showed significant inter-group distribution differences; the inter-group difference of *Coprostanoligenes* group reached a highly significant level (*P* = 0.0049), and its abundance increased slightly under specific soil conditioner treatments; the abundance of *Yersiniaceae* was lower in some single-agent treatments, with a significant inter-group difference (*P* = 0.03018); the relative abundance of *Hyphomicrobiaceae* remained around 1% across all treatments, with a large amplitude of fluctuation among treatments. At the fungal community level (Fig. 7B), inter-group differences were also prominent: the average relative abundance of *Cladosporiaceae* exceeded 5% under NM treatment, significantly higher than in other treatment groups; *Filobasidiaceae* showed obvious enrichment under NC treatment, with the highest value among all treatments; the relative abundance of *Chaetosphaeriaceae* ranged from 1.5% to 2% in most treatments, and decreased slightly under only one soil conditioner treatment; the inter-group difference of *Strophariaceae* was significant (*P* = 0.030), with its relative abundance being relatively prominent under biochar treatment; the abundance of *Helotiaceae* in the control treatment was slightly higher than that in the soil conditioner treatment groups, while *Tremellaceae* was more concentrated in some combined application treatments.

### Correlations between microbial community and environmental factors and their mediated pathways regulating yield

**Fig. 8:**
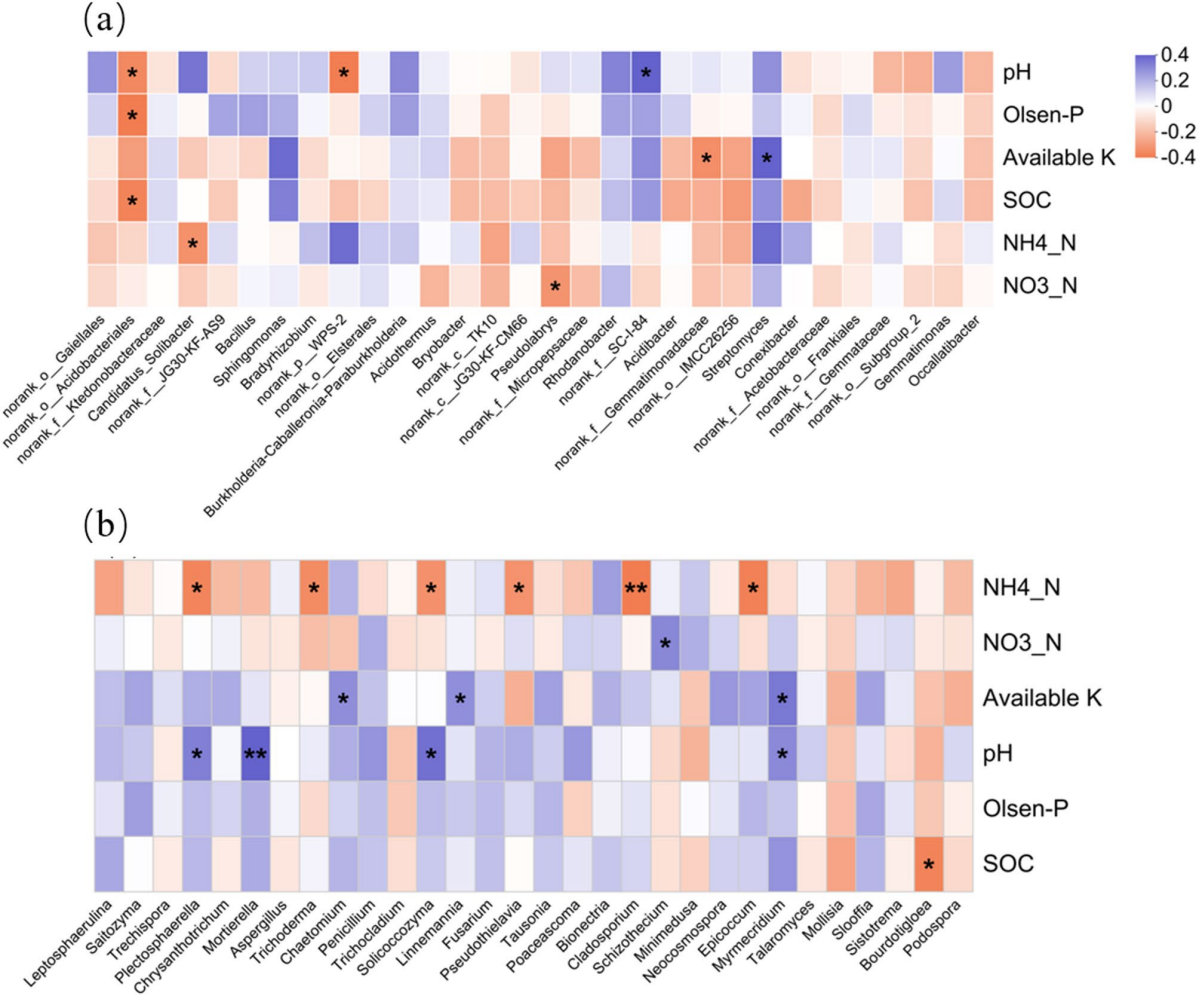
Correlation between soil properties and bacterial (A) and fungal (B) taxa. Spearman’s rank correlation coefficients and their corresponding *p*-values were calculated based on comparisons between the top 30 genera with relative abundance and soil properties. * and ** indicate significance at *P* < 0.05 and *P* < 0.01, respectively. Olsen-P (soil available phosphorus), Available K (soil available potassium), NH_4_^+^-N (soil ammonium nitrogen), NO_3_^−^-N (soil nitrate nitrogen), and SOC (soil organic carbon).

To clarify the intrinsic link between soil physical and chemical properties and microbial communities, this study generated a Spearman correlation matrix based on the top 30 microbial genera with the highest abundance. Bacterial community analysis (Fig. 8A) showed that pH, available phosphorus (AP), and organic carbon (OC) were significantly negatively correlated with unclassified *Acidobacteriales* (*norank_o__Acidobacteriales*) (*P* < 0.05); pH was negatively correlated with unclassified *WPS-2* (n*orank_p__WPS-2*) and positively correlated with unclassified SC-I-84 unclassified *Gemmatimonadaceae* (*norank_f__Gemmatimonadaceae*) was extremely significantly negatively correlated with *Streptomyces* (*P* < 0.01) and positively correlated with available potassium (AK). Fungal community analysis (Fig. 8B) showed that ammonium nitrogen (NH₄⁺-N) was significantly positively correlated with *Solicoccozyma*, *Plectosphaerella*, *Trichoderma*, *Epicoccum*, and *Pseudothielavia* (*P* < 0.05), and extremely significantly positively correlated with *Cladosporium* (*P* < 0.01); pH was negatively correlated with *Myrmecridium*, *Solicoccozyma*, and *Plectosphaerella* (*P* < 0.05), and extremely significantly negatively correlated with *Mortierella* (*P* < 0.01).

**Fig. 9:**
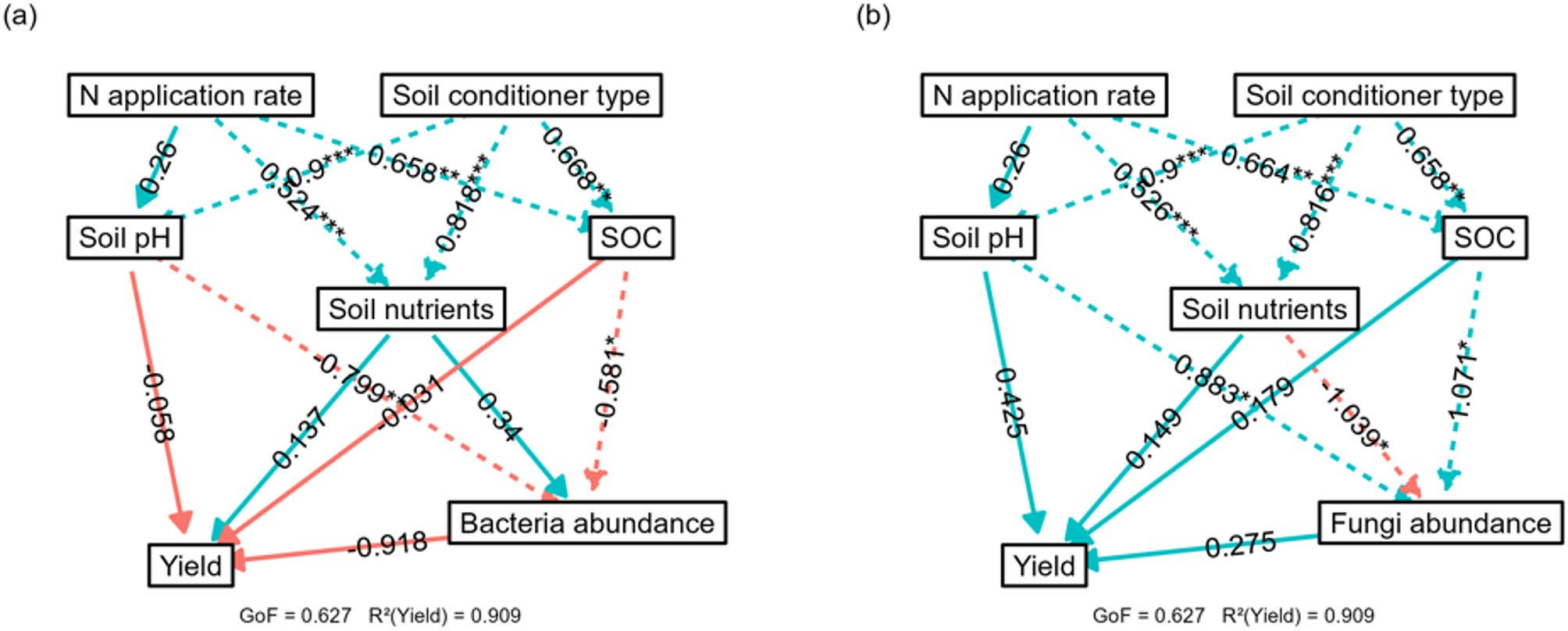
Partial Least Squares Path Modeling (PLS-PM) showing the effects of soil conditioners and nitrogen application on wheat yield. (a) represents the bacterial path model, and (b) represents the fungal path model. Red and green lines denote negative and positive correlations, respectively. Solid and dashed arrows indicate non-significant (*p* > 0.05) and significant (*p* < 0.05) paths, respectively. Values adjacent to the arrows are standardized path coefficients. Goodness-of-fit (Gof) statistics for the model are shown below the diagrams. SOC, soil organic carbon.

Partial Least Squares Path Modeling (PLS-PM) showed that nitrogen application rate and soil conditioner type jointly affected wheat yield by regulating soil physicochemical properties and microbial communities, with significant differences in the action pathways between bacterial and fungal communities (Fig. 9). Both models had a goodness-of-fit (GoF) of 0.627 and explained 90.9% of the yield variation (R²(Yield) = 0.909), indicating satisfactory overall model fit and strong explanatory power for yield formation mechanisms.

In the bacterial model, nitrogen application rate and soil conditioner type had highly significant positive direct effects on soil nutrients and soil organic carbon (SOC) (*P* < 0.001), laying the foundation for soil environment improvement. Further analysis revealed that soil pH and SOC exerted significant negative direct effects on bacterial abundance (*P* < 0.05), with path coefficients of − 0.799 and − 0.581, respectively. These results suggested that soil conditioners combined with nitrogen application could increase soil pH and SOC content, thereby reducing bacterial abundance and releasing its inhibitory effect on yield formation, ultimately leading to yield improvement.In contrast, the pathways in the fungal model differed substantially. Nitrogen application and conditioner treatments also significantly increased soil nutrients and SOC content (*P* < 0.001). However, SOC had a significant positive direct effect on fungal abundance (1.071, *P* < 0.05), whereas soil nutrients had a significant negative effect on fungal abundance (− 1.039, *P* < 0.05). Meanwhile, fungal abundance had a positive direct effect on yield (0.275), indicating that conditioner treatments promoted fungal community growth by increasing SOC content and thus directly enhanced yield formation. In addition, the direct positive effect of soil pH on yield (0.425) was more prominent in the fungal model, further reflecting the synergistic response between fungal communities and soil environment.

## Discussion

### Regulatory effects of soil conditioners on agronomic traits of wheat

Soil is the direct source of nutrients for crop growth. The application of different soil amendments can directly affect crop development by altering soil nutrient availability^11^. Two-year field data analyzed by two-way ANOVA showed that nitrogen application rate, amendment type, and their interaction (N×C) had highly significant effects on wheat grain yield and aboveground biomass (*P* < 0.001), indicating that the two factors did not function independently but exhibited synergistic regulation: amendment type regulated nitrogen use efficiency, while nitrogen supply also influenced the amelioration effect of amendments (Table 3). Further analysis of yield components revealed functional differentiation: spike number per unit area was significantly affected by nitrogen rate, grain number per spike was mainly regulated by amendment type, and their interaction had a highly significant effect on thousand-grain weight. As confirmed by Wu Mengjie et al.^12^, amendments such as lime and silicon-magnesium fertilizers can increase soil base cation content and thus enhance crop biomass. In addition, microbial fertilizer treatments also increased yield, suggesting that microbial inoculants can improve soil fertility and boost crop yield by introducing specific functional strains. This is because they significantly increase the abundance of nitrogen-fixing bacteria and functional groups involved in organic matter decomposition, whose metabolic activities help regulate soil acid-base environment and reduce acidity, thereby synergistically enhancing soil biological activity and promoting crop yield^13^. The NB (biochar + urea) treatment exhibited an excellent multi-pathway synergistic mechanism: chemically, biochar, rich in alkaline functional groups and stable carbon structure, can adsorb soil cations and buffer pH^14^; physically, the porous structure of biochar significantly improves soil aggregation and porosity, creating a favorable environment for root growth and development^15,16^.

**Fig. 10:**
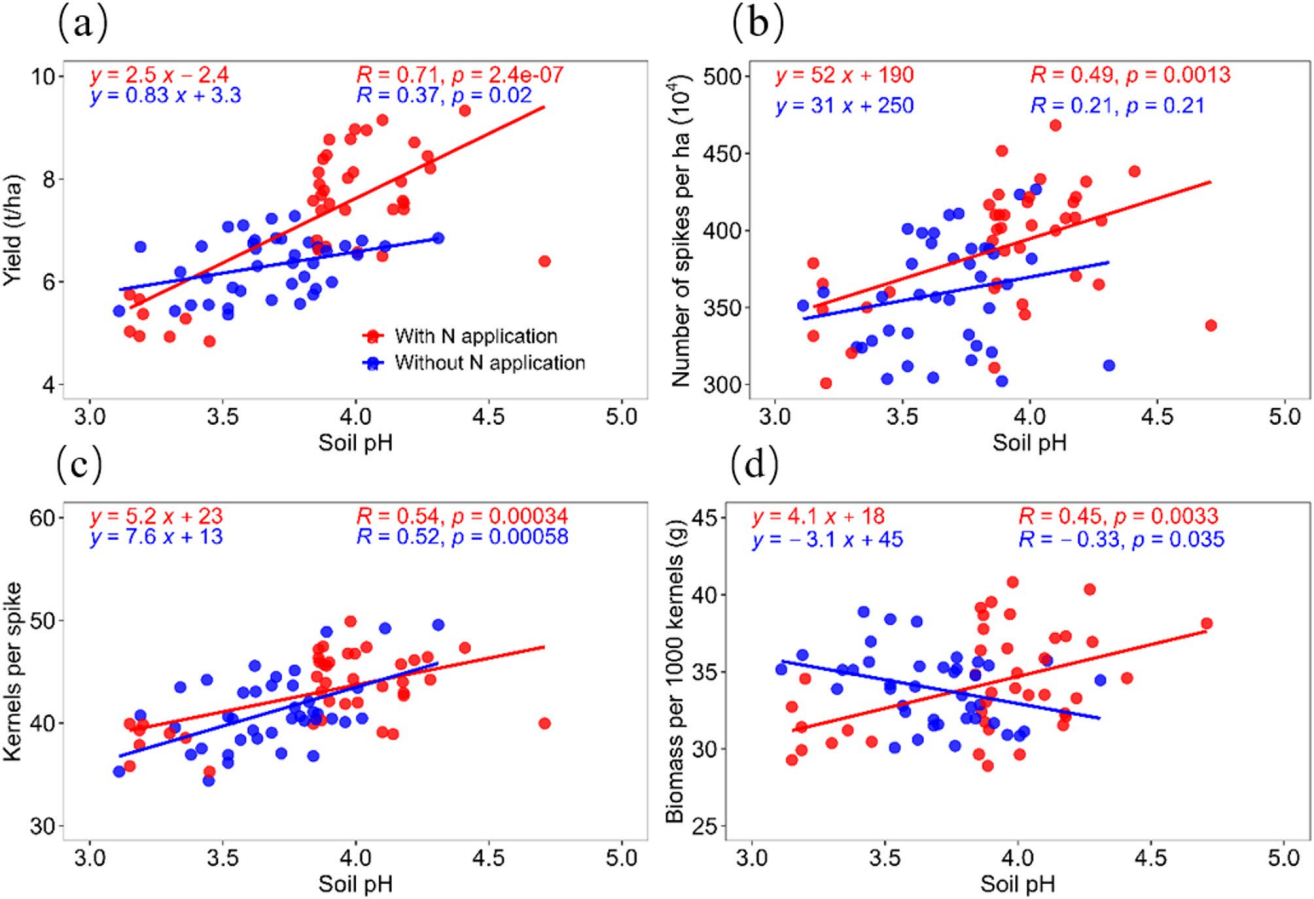
The relationship between soil pH and wheat yield (t ha^− 1^) with or without N fertilizer application.

**Fig. 11:**
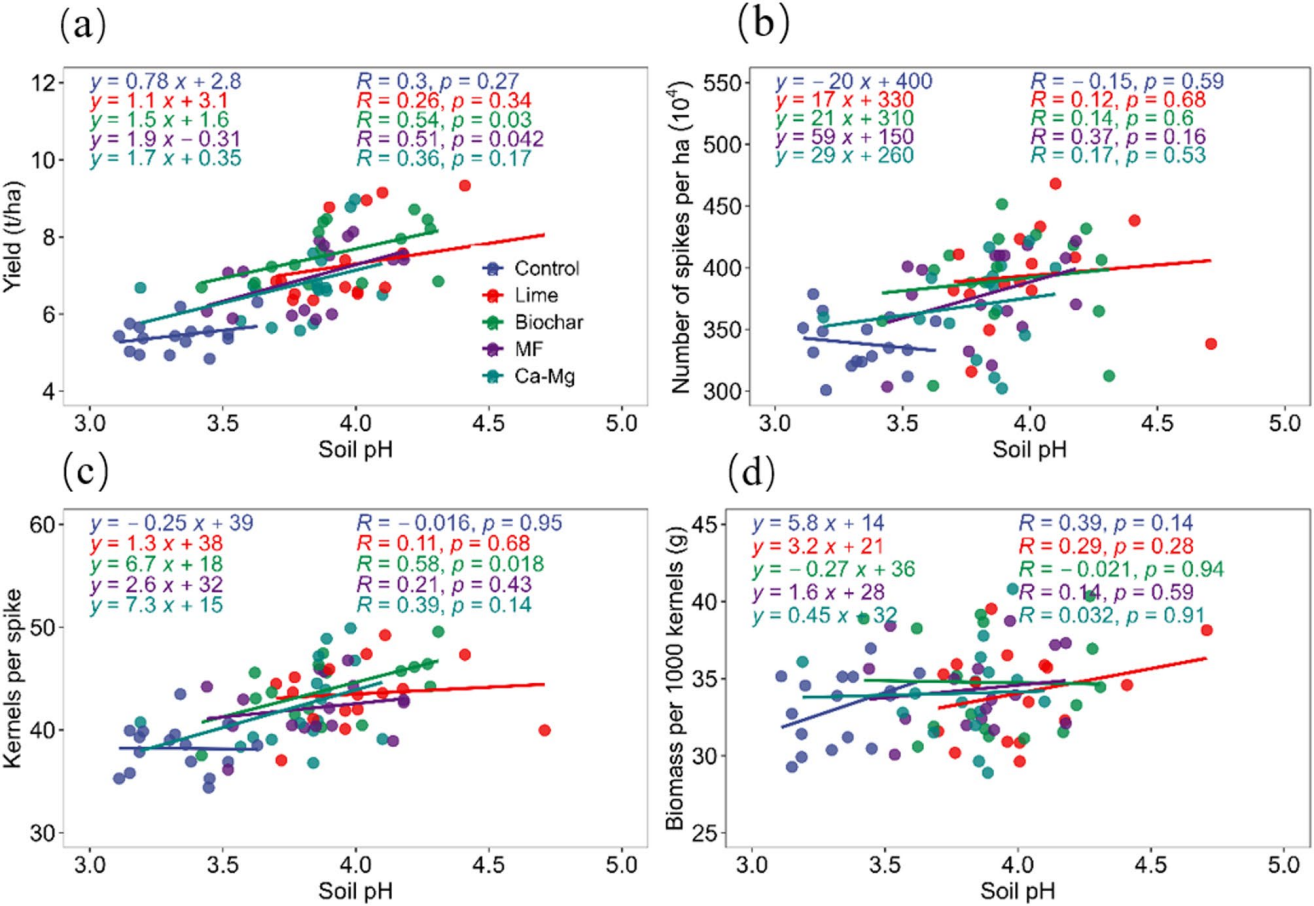
The relationship between soil pH and wheat yield (t ha^− 1^) with different soil conditioners application.

Further analysis of soil pH and its relationships with crop yield, spike number, grain number per spike, and thousand-grain weight showed distinct patterns under different nitrogen conditions. Under nitrogen application, soil pH was strongly positively correlated with crop yield (Fig. 10), and yield increased significantly with rising pH. Similarly, spike number, grain number per spike, and thousand-grain weight were all positively correlated with soil pH under nitrogen fertilization. These results indicate that increasing soil pH via amendments may enhance nitrogen use efficiency and thus promote high crop yield, consistent with the findings of Xia et al.^17^, because acid-reducing amendments significantly increase soil pH, crop yield, and partial factor productivity of nitrogen. In contrast, without nitrogen application, the correlations between soil pH and crop growth indices were much weaker. Although yield, spike number, and grain number per spike still showed positive trends, the correlations were weaker than under nitrogen application; notably, thousand-grain weight was negatively correlated (Fig. 11). This suggests that soil pH affects crop growth, but its effect is more pronounced under sufficient nitrogen supply, highlighting the synergy between pH regulation and nitrogen fertilization in optimizing crop yield and quality. The relationship between soil pH and wheat yield varied among amendments: lime and biochar treatments showed stronger correlations, with lime treatment significantly increasing yield with rising pH compared to the control^18^, whereas correlations were weaker in the control and other treatments.

### Improvement effects of soil conditioners on physicochemical properties of acidic soils

Long-term excessive fertilization in wheat cultivation has led to severe soil acidification, significantly inhibiting crop growth and threatening agricultural sustainability. Suitable soil amendments can neutralize acidic ions by releasing alkaline groups, thereby increasing soil pH and promoting crop growth^19^. The results of this study indicate that both the lime-urea combined treatment (NL) and the biochar-urea combined treatment (NB) significantly outperformed the control in raising the pH of acidic soil. This is because lime can continuously react with soil acids, resulting in longer-lasting amelioration effects^20^, while biochar increases the specific surface area of soil colloids, enhances adsorption capacity, and promotes the retention of more cations on colloid surfaces^21^. Compared to the application of single soil amendments (lime or biochar), these combined amendments not only effectively promote urea assimilation and soil nutrient release but also further optimize nutrient availability, creating more favorable conditions for crop nutrient uptake and further increasing soil pH.

Adequate supply of available nutrients, particularly during the early growth stage, is crucial for seedling root establishment and photosynthetic system development, directly influencing final yield and quality. The nutrient demand during this phase is high, and deficiencies can easily limit yield potential. As a classic amendment for acidic soils, lime releases alkaline ions to neutralize soil acidity and raise pH. This not only enhances potassium ion activity but also activates potassium-related functional microorganisms, thereby supporting early potassium uptake by crops^22^. The combined application of biochar and urea (NB) showed more comprehensive advantages in nutrient regulation and soil improvement, and significantly increased soil available potassium (AK), organic carbon (OC), ammonium nitrogen (NH₄⁺-N) and nitrate nitrogen (NO₃⁻-N) contents (*P* < 0.05). Results from the two-year long-term experiment indicated that although soil NH₄⁺-N and NO₃⁻-N contents under the NB treatment were slightly lower than those under the sole urea application, they were significantly higher than those in the control and single amendment treatments, maintaining a high level of soil available nitrogen and ensuring nitrogen supply for wheat during the growth period. This was partly attributed to the fact that biochar retained soluble phosphate formed during its production process, which directly increased soil available phosphorus content after being applied into the soil^23^. Additionally, biochar application raises soil pH, thereby influencing phosphorus transformation and availability^24^. Moreover, due to its strong adsorption capacity, biochar enhances soil retention of organic matter, adsorbs organic molecules, and promotes their polymerization, thereby increasing soil organic matter content, which aligns with the findings of Xiang Shujiang et al.^25^. Aggregate fractionation analysis further confirmed its carbon sequestration advantage: under the NB treatment, the organic carbon content in both < 0.25 mm micro-aggregates and > 2 mm macro-aggregates increased compared to the control. Stable aggregate structure not only improves soil aeration and reduces compaction risk but also provides a looser physical environment for wheat root penetration, further enhancing nutrient uptake efficiency^26^.

### Restructuring effects of soil conditioners on microbial communities in acidic soils

Soil microbial diversity is an important indicator for evaluating soil ecological functions. Compared with physicochemical indicators, it can rapidly reflect changes in soil quality and serves as the foundation for maintaining ecosystem stability^27^. This study found that compound conditioners such as lime, NB, and NC can effectively optimize the microbial community structure in the wheat rhizosphere in acidic soils. By analyzing changes in rhizosphere microbial α-diversity and dominant taxa, we confirmed the ameliorative effect of conditioners on the microbial habitat. This result is consistent with the report by He et al.^28^, which showed that biochar application increases soil cumulative porosity and pore surface area density, promotes the transformation of compact blocky soil structure into a porous structure (macropores and mesopores), provides a suitable living environment for microorganisms, and thereby enhances microbial richness and diversity. Further analysis revealed significant correlations between soil physicochemical properties and microbial taxa. Both nitrogen application rate and conditioner type jointly drive changes in physicochemical properties and influence crop yield through different pathways—bacterial communities show a negative effect, while fungal communities exhibit a positive effect. This reveals the potential mechanism by which conditioners indirectly affect yield through the regulation of microbial community structure. Soil conditioners can directly or indirectly reshape the structure and function of soil microbial communities by regulating soil physicochemical properties (such as pH, nutrient availability, and organic matter content)^29^.

### Analysis of the correlation between microbial community structure and environmental factors and the pathway of yield regulation

This study found that bacterial and fungal communities exhibited significantly different responses to soil conditioner treatments. Analysis of the effects of different soil improvement measures on soil microbial communities revealed marked differences in the response patterns of bacterial and fungal communities: bacterial communities were more sensitive to combined treatments (e.g., NB treatment), with a significant increase in the relative abundance of their dominant taxa (e.g., *Rhodanobacteraceae* and *Gaiellales*); in contrast, fungal communities showed relatively conservative responses, exhibiting stronger ecological stability and being less influenced by soil physicochemical properties^30^. Previous studies have also confirmed that fungi are generally more conservative than bacteria in response to soil management practices, and this difference may be closely related to their unique ecological strategies and physiological characteristics^31^. Meanwhile, based on Spearman correlation analysis, this study further clarified the significant associations between soil physicochemical properties and specific microbial taxa. Specifically, pH was significantly negatively correlated with *Acidobacteriales*, which is consistent with previous reports that the phylum *Acidobacteriota* often dominates in acidic soils^32^; ammonium nitrogen (NH₄⁺-N) was significantly positively correlated with beneficial fungi such as Trichoderma, suggesting that nitrogen supply may favor the colonization of antagonistic fungi^33^.

Furthermore, based on Spearman correlation analysis and PLS-PM, this study further elucidated the significant associations between soil physicochemical properties and specific microbial taxa: pH was significantly negatively correlated with *Acidobacteriales*, consistent with previous reports that the phylum *Acidobacteriota* often dominates in acidic soils^32^; while ammonium nitrogen (NH₄⁺-N) was significantly positively correlated with beneficial fungi such as Trichoderma, suggesting that nitrogen supply may favor the colonization of antagonistic fungi^33^. PLS-PM analysis further revealed the differential regulatory pathways of bacteria and fungi on wheat yield. In the bacterial model, both pH and soil organic carbon (SOC) exerted significant negative effects on bacterial abundance (path coefficients of -0.799 and − 0.581, respectively), indicating that combined conditioners can inhibit the excessive proliferation of certain bacteria (especially acid-adapted taxa) by increasing soil pH and SOC content, thereby alleviating their inhibitory effect on yield formation^34,35^. In the fungal model, SOC had a significant positive effect on fungal abundance (path coefficient 1.071), and fungal abundance further positively affected wheat yield (path coefficient 0.275), while pH also had a direct positive effect on yield (path coefficient 0.425), indicating that conditioners can promote fungal community growth by increasing SOC content, thereby directly enhancing wheat yield^36^. Notably, recent studies have confirmed that the combined application of biochar and nitrogen fertilizer can significantly increase crop yield by optimizing soil physicochemical properties and microbial community structure^37^, which is consistent with the findings of this study.

## Conclusion

The study demonstrates that soil conditioners, particularly composite treatments like NB, significantly improve soil properties and wheat growth in acidic soils. These conditioners enhance soil pH, nutrient availability, and microbial diversity, promoting higher wheat yields and better nutrient uptake. Lime and biochar treatments showed notable effects on soil structure and carbon sequestration, while the NB treatment outperformed others in enhancing both physical and chemical soil conditions. The research underscores the importance of integrated soil improvement strategies in optimizing crop production and soil health, highlighting the synergistic benefits of combining soil conditioners with nitrogen fertilization. Furthermore, microbial community analysis revealed that soil pH and nutrient levels strongly influenced microbial composition, offering insights into the role of microbial dynamics in soil fertility and crop performance.

## Perspectives

This study had a two-year experiment period, which is insufficient to capture the long-term effects of conditioners (e.g., biochar, microbial fertilizer) on soil carbon pools and microbial communities. Moreover, the study focused only on wheat without incorporating the typical wheat–maize rotation system in southern Henan, limiting the generalizability of the conclusions. Future studies should implement long-term field experiments, include the rotation system, and expand to more regions and soil types to further validate the applicability of the conditioners, thereby enhancing the scientific rigor and generalizability of the findings.

## Data Availability

The datasets used and/or analyzed during the current study are available from the corresponding author upon reasonable request.
